# Gender dynamics in drug-using contexts: Implication for treatment and support

**DOI:** 10.1177/14550725251328217

**Published:** 2025-03-25

**Authors:** Anne Schanche Selbekk, Inger Eide Robertson, Espen Enoksen, Silje Gundersen, Nina Grashei, Kristin Sanne Mohn, Bente Sikveland

**Affiliations:** Center for Alcohol and Drug Research (KORFOR), 60496 Stavanger University Hospital, Stavanger, Norway; Department of Public Health, University of Stavanger, Stavanger, Norway; KORUS, 60496 Stavanger University Hospital, Stavanger, Norway; Center for Alcohol and Drug Research (KORFOR), 60496 Stavanger University Hospital, Stavanger, Norway; A-larm, Stavanger, Norway; Department of Substance use and Addiction Treatment, 60496 Stavanger University Hospital, Stavanger, Norway

**Keywords:** addiction, drug-using context, gender dynamics, substance use, trauma, treatment, women

Gender has been a recurring theme in discussions on addiction and substance use problems. Evidence indicates biological, psychological, relational and social differences in the use of substances and the development of substance-related problems between genders, drawing on research from both an epidemiological and a first-person perspective (Campbell & Herzberg, 2017; Chang, 2023; Hunt et al., 2015; McHugh et al., 2018). The gender perspective arises in various ways, manifested through different preferences for substances and diverse societal norms regarding intake and substance-related behaviour and a different risk-environment (Mayock et al., 2015). Data from the Norwegian KVARUS register (National Quality Register for the treatment of harmful use of substances or addiction) (Kvalitetsregistre, 2019) are pinpointing a distinct “gendered” profile in the way that patients have experienced traumatic experiences in their life. Both men and women have largely experienced exposure to and witnessing of violence (70–80%). However, compared to men, women have more frequently experienced neglect during their upbringing (51% vs. 38%), as well as sexual violations and abuse (60% vs. 14%), and have been in destructive relationships (63% vs. 42%). Conversely, men have more often exposed others to violence compared to women (45% vs. 29%). Similar results are found in international research (Marshall et al., 2008; Mayock et al., 2015). Illegal drug-using contexts is known for its violence and brutality, and the connection between illicit drug use and violence is well recognized (Copes et al., 2015; Grundetjern, 2015). Furthermore, the illegal drug using culture is traditionally described as being male dominated, even though some studies report a growing number of female drug dealers (Grundetjern, 2015; Grundetjern & Sandberg, 2012). Street-based drug scenes has been identified with a high concentration of people at the margins of society (McNeil et al., 2014), characterized by brutalities and gender-based hierarchies (Copes et al., 2015). So, what are the dynamics fuelling the differences between men and woman? We still lack knowledge on the life of people living in illegal drug using cultures in general (Johansen, 2025) and, more specifically, the differences between men and women. And in what way can and should our services on different levels take this knowledge into account? The historical gap between men and women in the prevalence of substance use disorders internationally, with a higher prevalence among men, is narrowing (McHugh et al., 2018). In alcohol and other drug treatments, there has been an overrepresentation of men in patient groups, alongside a predominance of female clinicians. There are indications that women are less likely to enter treatment compared to men (Greenfield et al., 2007). It is emphasized that we lack knowledge about women's needs and experiences related to gender-specific treatment, just as we need more focus on men's unique needs in the follow-up and treatment of substance-related problems, and similarly in relation to queer gender identity. National guidelines in Norway conclude that gender-specific treatment and follow-up needs should be addressed in the course of treatment (Helsedirektoratet, 2016).

The issue of gender and problematic substance use and addiction formed the groundwork for an annual dialogue conference, arranged by the Stavanger Group for Alcohol and Drug research (STARUS) on “Gender, substance use and addiction” in Stavanger, Norway, April 2023. The dialogue conference, with approximately 90 participants, included people with user experience, clinicians, managers and researchers in the region. Using Open Space, a dialogue-based methodology (Svendsen et al., 2012), the content of the conference is based on the subthemes suggested by the participants. Based on utterances and statements abstracted from the many group discussions, we found some interesting issues and aspects relevant to take forward for debate and exploration.

## Gender-related dynamics in drug-using economies and contexts

First, in the groups, there were discussions regarding how brutality and violent episodes were a part of life in illegal drug-using contexts for all the persons involved, including both men and women, aligning with the data on exposure to trauma. At the same time, it was emphasized how many women are exposed to trauma differently from men, and that these traumas are often related to gender dynamics, where women are subjected to abuse from men and fear them. Consequently, women seek protection from a man selling drugs and become dependent on them. Some participants expressed the notion that women are “owned” by someone, and how the complex interplay of love and exploitation further complicates their position. Women often find themselves at the bottom of the hierarchy, and it was also reflected on in the discussion that woman punished themselves the most. Still, it was also emphasized how this varied a lot from woman to woman, with some seeking independence and refusing to be a victim, while others seek protection.

Futhermore, in the group discussions, it was highlighted how drama, intrigues and conflicts between women were pointed out as a common phenomenon related to drug-using contexts. This “drama” between women was spoken about in relation to women fighting over crucial positions in the male dominated hierarchies for own safety and protection. It is reasonable to assume that these “attractive” male positions are few in number and hard to get and keep, resulting in tough competition between female persons who use drugs, narrated as “lots of drama between women”. Thus, women in illegal drug-using contexts constantly need to ensure their own safety and protection through fighting for a position related to a male person dealing drugs with a high standing in the drug-using economy (Grundetjern & Sandberg 2012). However, it might be argued that the above explanations portray women in illegal drug-using economies and contexts as victims and male-dependent. As an alternative explanation, “the drama” between females might relate to strategies putting them in more powerful positions, as Grundetjern and Sandberg (2012) show in their studies of female drug dealers. Being embedded in a male dominated drug-using culture generates gendered strategies for women to overcome and cope with this disadvantage (Grundetjern & Sandberg, 2012). It can also be seen as men utilizing status and women utilizing sex and caring to survive. But, in this, women protect or defend men more than they do other women.

Another gender-related aspect that was raised in the discussion is how women often assume significant relational responsibilities within the social environment. They take on a caretaker role, and some women find themselves staying longer in certain situations due to this responsibility. Unfortunately, this sense of responsibility can also make it more challenging for them to break free or exit from such circumstances.

## Challenges faced when seeking out assistance

Second, how does these dynamics play out when entering new social arenas, like services or treatment? Based on utterances and statements in the discussions, women seem sceptical to gender-specific treatment. A concrete example was related to female group therapy. This scepticism was justified in wanting to avoid “the female drama”. Participants also expressed how being in a social context with only women (e.g., in group therapy) was something “new”. Women look at you in a different way than a man, and you cannot relay to the dynamics of sex and caregiving. They discussed how women often feel apprehensive about such situations because they are unfamiliar, especially if they come from environments with few female friends. Interestingly, female participants at the conference, who had firsthand experience with AOD (Alcohol and Other Drug) treatment, described a process in which female groups eventually became a crucial aspect of their treatment. As they opened and shared their experiences, they found support in each other, through therapy, by helping each other in setting boundaries and as inspirators and role models. This allowed them to forge friendships with other women, moving beyond mere competition. These processes are closely tied to the challenges and dilemmas of leaving behind an old environment while being aware that the risk of relapse still exists. The cost of severing ties with established positions and alliances in the drug-using context is a significant consideration. During discussions, participants acknowledged that people often revert to what feels safe and familiar, even within the treatment setting, where patterns from active drug use may persist. It was also uttered how the level of experienced safety needs to be so high in a treatment facility that people dare to seek treatment in the first place.

Other stories abstracted from the group discussions were related to the phenomenon that women tend to establish intimate partnerships within the inpatient treatment facilities for protection, but maybe also for securing personal safety after discharge, which also could result in drop-out, relapse and continuing drug use. Forming a relationship with someone in treatment can also serve as a substitute for substances, with romantic relationships being a second-best option. Since establishing relationships within treatment is unwanted from an institutional perspective, these relationships are kept hidden. Everyone knows about them, but there is no space to discuss the dynamics involved. These dynamics might obstruct successful treatment. Narratives were also shared about how women can experience abuse in the context of treatment. Some women may feel hesitant about entering an environment where there is an overrepresentation of male patients.

In a recovery perspective, it is also interesting to consider how changes can lead to a transition into a social context with different gender dynamics, where gender equality becomes the norm and standard. Additionally, the shift from a former life in a drug-using economy, experienced by both men and women, represents a significant transition in this regard.

## Challenges to bring forward

The results from the group discussion at the conference highlight some important challenges to bring forward.

There are important links between patterns of social interaction in open drug scenes on the one hand and patterns of social interaction and conditions of possibility to achieve wanted changes within a treatment or service context on the other. To make treatment or services, as well as follow-up after discharge, relevant and useful, those links must be taken into consideration. To our knowledge, this link has been the subject of limited research, and there seems to be a lack of awareness, attitude and understanding among the staff in the services regarding the importance of gender and gender-specific considerations.

Therefore, we propose a research agenda to explore how insights into gender dynamics within drug-using economies and contexts can inform and reshape support systems, ensuring impactful responses to the complex challenges faced by those seeking assistance.

First, we need to know more in-depth about: (1) What is distinct about gender and gender identity related to drug use and the social contexts in which this use occurs? (2) What do we know about how gender identities manifest in different user environments? (3) What roles and positions are expected for “men” and “women” in substance use context and how are they changing over time? (Typical “women” as victims and “men” as dominant abusers). (4) What gender role patterns are produced in (different) illegal drug use contexts?

Furthermore, we need to gain a deeper understanding of how these characteristics play out in treatment and service contexts. Exploring different formats, such as sequential treatments and gender-specific programs, is essential. For example, how are social interactions within drug-using contexts addressed or thematized in in-patient treatment settings?

Considerations should also extend to how gender roles and violence are discussed and integrated into treatment. Is there a prevailing “silence knowledge” around these topics? It is crucial to focus not only on the psychological aspects, but also on the social dynamics.

Collaboration between men and women is essential. What can they work on together, and what areas require gender-specific attention? Identifying gender-specific treatment needs, especially related to trauma, is vital. The overall question is: how can we effectively address these needs?
